# Trends in the Frequency of Severe Maternal Morbidity Among American Indian and Alaska Native Individuals at Two Major Hospitals in the East Valley, Arizona

**DOI:** 10.7759/cureus.116421

**Published:** 2026-09-17

**Authors:** Abigail J Nielsen, Noor Farghaly, Laila Younes, Jane Ahn, Abdul Waheed, Erum Azhar

**Affiliations:** 1 Department of Obstetrics and Gynecology, Creighton University School of Medicine, Phoenix, USA; 2 Department of Obstetrics and Gynecology, Dignity Health East Valley, Obstetrics and Gynecology Residency Program, Gilbert, USA; 3 Department of Family Medicine, Dignity Health, Gilbert, USA; 4 Department of Family Medicine, Creighton University East Valley, Family Medicine Residency Program, Gilbert, USA; 5 Department of Family and Community Medicine, Creighton University School of Medicine, Phoenix, USA

**Keywords:** alaska native, american indian, arizona, health disparities, severe maternal morbidity

## Abstract

Introduction

The United States continues to see rising rates of maternal mortality (MM) and severe maternal morbidity (SMM), with American Indian and Alaska Native (AI/AN) individuals facing particularly high risk. Arizona shows marked maternal health disparities, yet localized data on AI/AN maternal health remains limited. This study set out to examine trends in SMM among AI/AN birthing individuals in the East Valley of Arizona. By focusing on hospital-level data, the study aimed to address critical gaps in region-specific AI/AN maternal health data that are often underrepresented in statewide or national analyses. Understanding local trends is essential for identifying actionable disparities and shaping targeted interventions.

Methods

A retrospective cross-sectional analysis was carried out using de-identified data from Chandler Regional Medical Center (CRMC) and Mercy Gilbert Medical Center (MGMC) in the East Valley of Arizona, facilities that are part of the largest integrated healthcare system, between January 2020 and December 2025. Shewhart control charts were used to establish baseline SMM rates and identify racial disparities. The study population consisted of eligible delivery hospitalizations at CRMC and MGMC during the study period. Delivery hospitalizations were stratified by documented race and ethnicity, and SMM cases were identified within each racial and ethnic group. AI/AN birthing individuals served as the primary population of interest. Data were analyzed using JMP Pro 19 software. Linear regression, subgroup race and ethnicity analyses, and Statistical Process Control (SPC) charts were used to assess disparities and temporal trends. Analyses were designed to detect both between-group differences and within-group changes over time, with particular attention to identifying statistically significant trends in SMM rates.

Results

AI/AN birthing individuals recorded the highest cumulative SMM rate across all racial and ethnic groups analyzed at both hospitals (4.12%; 64 SMM cases among 1,554 deliveries). AI/AN individuals saw a more than fivefold increase in SMM rates, from 1.13% in 2020 to 6.17% in 2025, and were the only racial or ethnic group to show a statistically significant increase over time (0.848 percentage points per year; R² = 0.6866, p = 0.0415).

Conclusions

These findings highlight a growing maternal health disparity among AI/AN populations in the East Valley. Future analyses will include multivariate and subgroup comorbidity analyses, including age, prenatal care, comorbid conditions, and socioeconomic indicators. Local, community-informed interventions are urgently needed to address these worsening trends and improve maternal outcomes. Hospital-based, culturally responsive data analysis could serve as a model for identifying and addressing maternal health inequities in other regions with significant AI/AN populations.

## Introduction

The United States continues to confront rising maternal mortality (MM) rates, with disproportionate impacts on racially and ethnically marginalized populations [1,2]. American Indian and Alaska Native (AI/AN) individuals are among the most severely affected, facing some of the highest MM rates nationwide [1,3,4]. In Arizona, maternal health inequities are particularly pronounced. State-level reviews conducted between 2016 and 2019 found that AI/AN birthing individuals had severe maternal morbidity (SMM) rates more than 3.5 times higher than those of non-Hispanic White birthing individuals [5].

Clinical, healthcare access, and structural factors may contribute to these disparities. Cardiovascular disease is a leading cause of MM and SMM in the United States, with cardiomyopathy and hypertensive disorders of pregnancy standing out as particularly significant contributors to SMM among AI/AN populations [6,7]. AI/AN populations also experience higher rates of chronic conditions, including hypertension, obesity, diabetes, and kidney disease, which are associated with increased risk of pregnancy complications [8,9]. Structural and healthcare access barriers affecting AI/AN communities have also been identified, including geographic isolation, limited access to prenatal and obstetric care, and gaps in culturally appropriate care, all of which may contribute to maternal health disparities [10,11].

Despite these disparities, research on the maternal health of AI/AN birthing individuals remains limited. Given the geographic, cultural, and linguistic diversity among AI/AN populations, localized studies are essential to accurately capture maternal health outcomes and to guide future research and interventions. This study examines SMM stratified by race and ethnicity among birthing individuals in the East Valley of Arizona. The primary objectives of this study were to (1) compare SMM rates across racial and ethnic groups among birthing individuals receiving care at Chandler Regional Medical Center (CRMC) and Mercy Gilbert Medical Center (MGMC), with a particular focus on AI/AN individuals, and (2) evaluate temporal trends in SMM during the study period. Subsequent phases will include subgroup analyses by age, access to prenatal care, socioeconomic factors, and comorbid conditions. These future analyses could help identify factors associated with observed disparities and guide the development of targeted, culturally responsive interventions in collaboration with AI/AN community partners.

The abstract of this article was previously presented as a poster at the Institute for Healthcare Improvement Forum (December 8-9, 2025) and as an oral presentation at Creighton Research Day (March 31, 2026).

## Materials and methods

This retrospective cross-sectional study examined de-identified data from CommonSpirit Health, a large integrated healthcare system, to assess SMM among birthing individuals in the East Valley region of Arizona at two large hospitals, CRMC and MGMC, between January 2020 and December 2025. The primary objectives were to compare SMM rates across racial and ethnic groups and evaluate temporal changes in SMM over the study period, with a particular focus on AI/AN birthing individuals.

Data were extracted from the Cerner electronic health record system using the established analytics infrastructure of the CommonSpirit Health Population Health Department. All data were fully de-identified before analysis, and no names, medical record numbers, or other personal identifiers were retained. The study protocol was approved by the Dignity Health Arizona Regional Institutional Review Board (DHARE-IRB #00001993; protocol #EVR-25-510-324-73-47).

The study population consisted of eligible delivery hospitalizations at CRMC and MGMC between January 2020 and December 2025. SMM cases were identified using the Centers for Disease Control and Prevention (CDC) SMM criteria. Duplicate encounters associated with the same SMM event were excluded to avoid duplicate case counting. Delivery hospitalizations were stratified by documented race and ethnicity, with AI/AN birthing individuals serving as the primary population of interest. Delivery hospitalizations with missing, unknown, or declined race/ethnicity information were excluded from race/ethnicity-stratified analyses.

Statistical analyses were carried out using JMP Pro 19 software. All statistical tests were two-sided, and p < 0.05 was considered statistically significant. Descriptive statistics were used to summarize SMM outcomes. Statistical Process Control (SPC) analyses using individual values and moving range (I-MR) control charts were applied to evaluate monthly SMM case counts at CRMC and MGMC. SMM rates were calculated using the number of SMM cases within each racial and ethnic group as the numerator and the corresponding number of delivery hospitalizations as the denominator. Linear regression was used to evaluate temporal trends in annual SMM case counts at CRMC and MGMC and annual SMM rates among AI/AN individuals.

## Results

To establish a baseline understanding of SMM incidence, a rigorous SPC analysis was conducted across two major medical centers.

SMM case count trends by hospital

At CRMC, monthly SMM case counts were analyzed with an individual values and moving range (I-MR) control chart (Figure 1). The mean monthly SMM case count stood at 8.77 cases per month over the study period.

**Figure 1:**
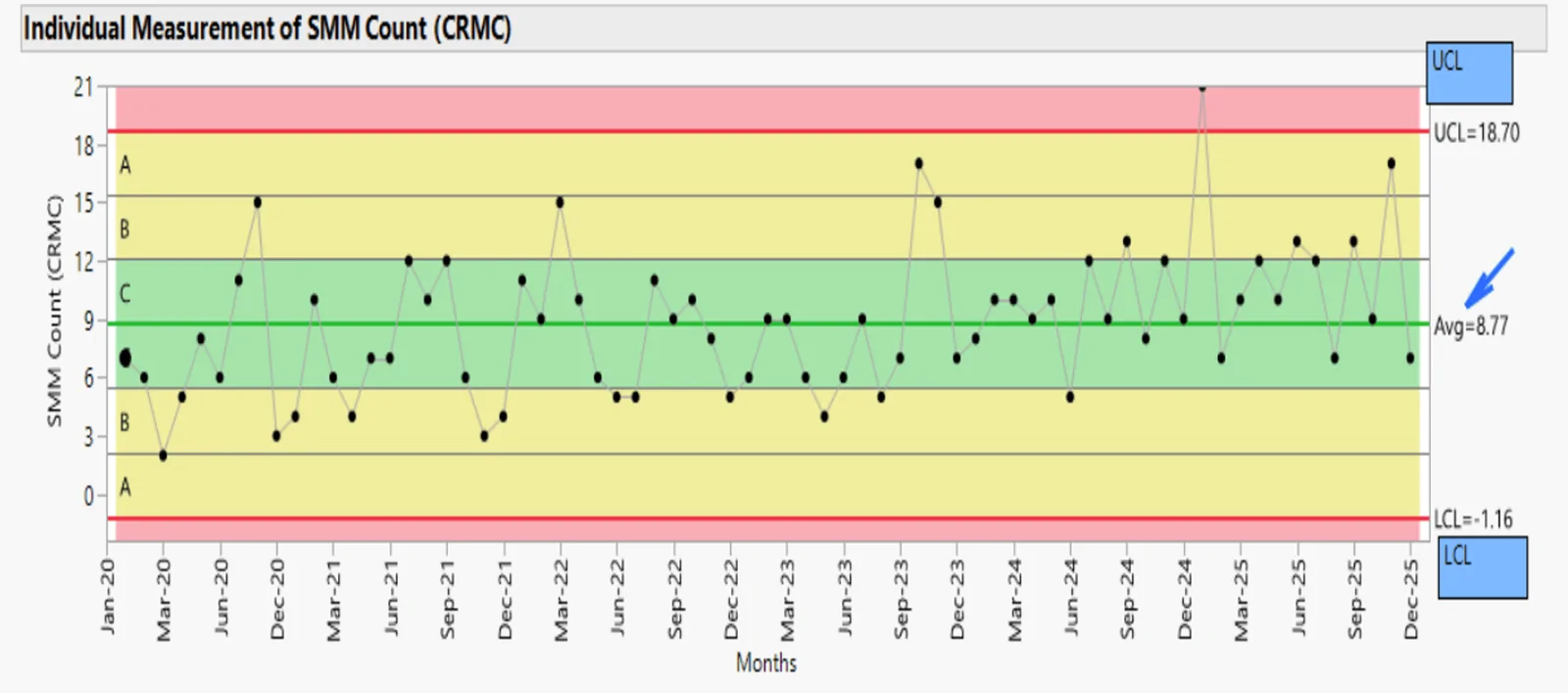
Individual values and moving range (X-MR) control chart of monthly SMM counts at CRMC The mean SMM count is 8.77 cases per month (blue arrow) SMM: severe maternal morbidity; CRMC: Chandler Regional Medical Center; UCL: upper control limit; LCL: lower control limit

Similarly, at MGMC, monthly SMM case counts were analyzed with an individual values and moving range (I-MR) control chart (Figure 2). The mean monthly SMM case count stood at 8.12 cases per month over the study period.

**Figure 2:**
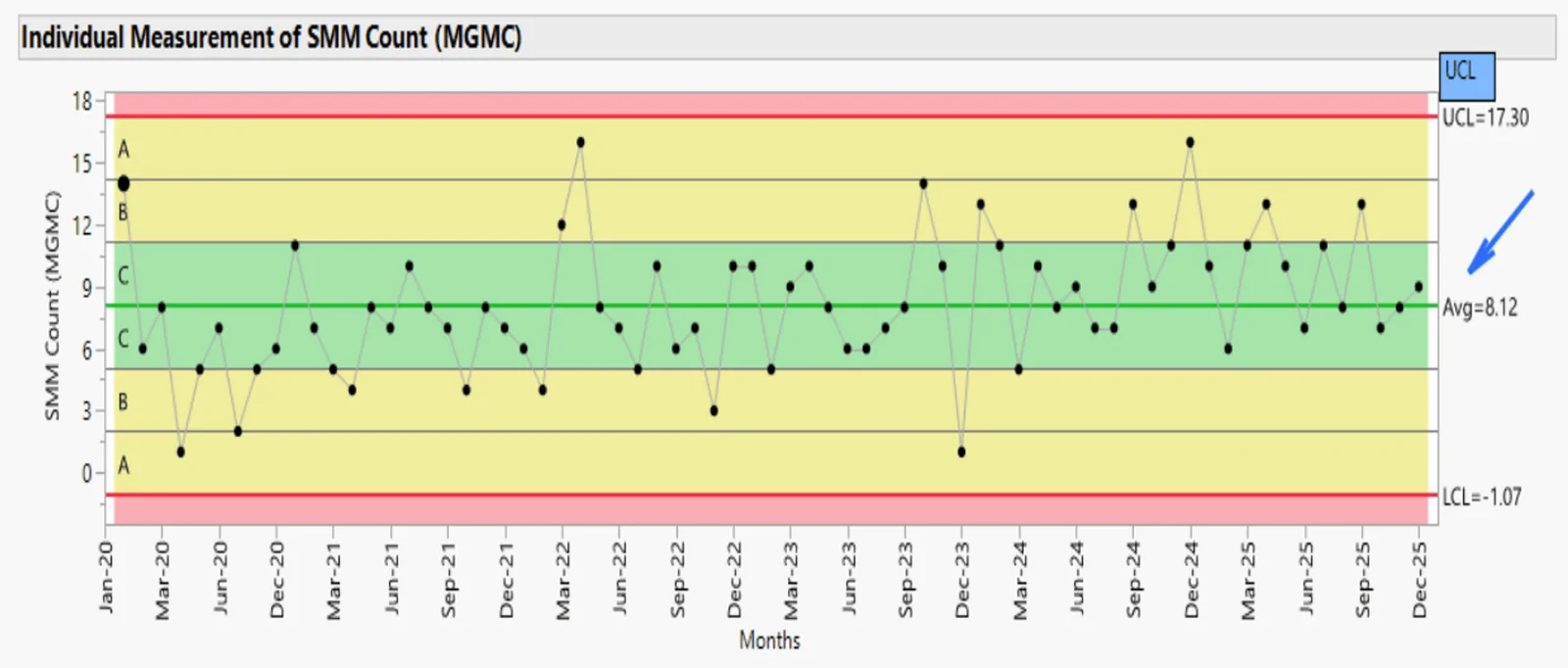
Individual values and moving range (X-MR) control chart of monthly SMM counts over time at MGMC The mean SMM count is 8.12 cases per month (blue arrow) SMM: severe maternal morbidity; MGMC: Mercy Gilbert Medical Center; UCL: upper control limit; LCL: lower control limit

As shown in Figure 3, linear regression demonstrated increasing SMM case counts over time at both CRMC and MGMC. At CRMC, SMM cases increased by 12.6 per year, while at MGMC, cases increased by 10.9 per year; both trends were statistically significant (CRMC: R² = 0.93, p = 0.0019; MGMC: R² = 0.81, p = 0.0142).

**Figure 3:**
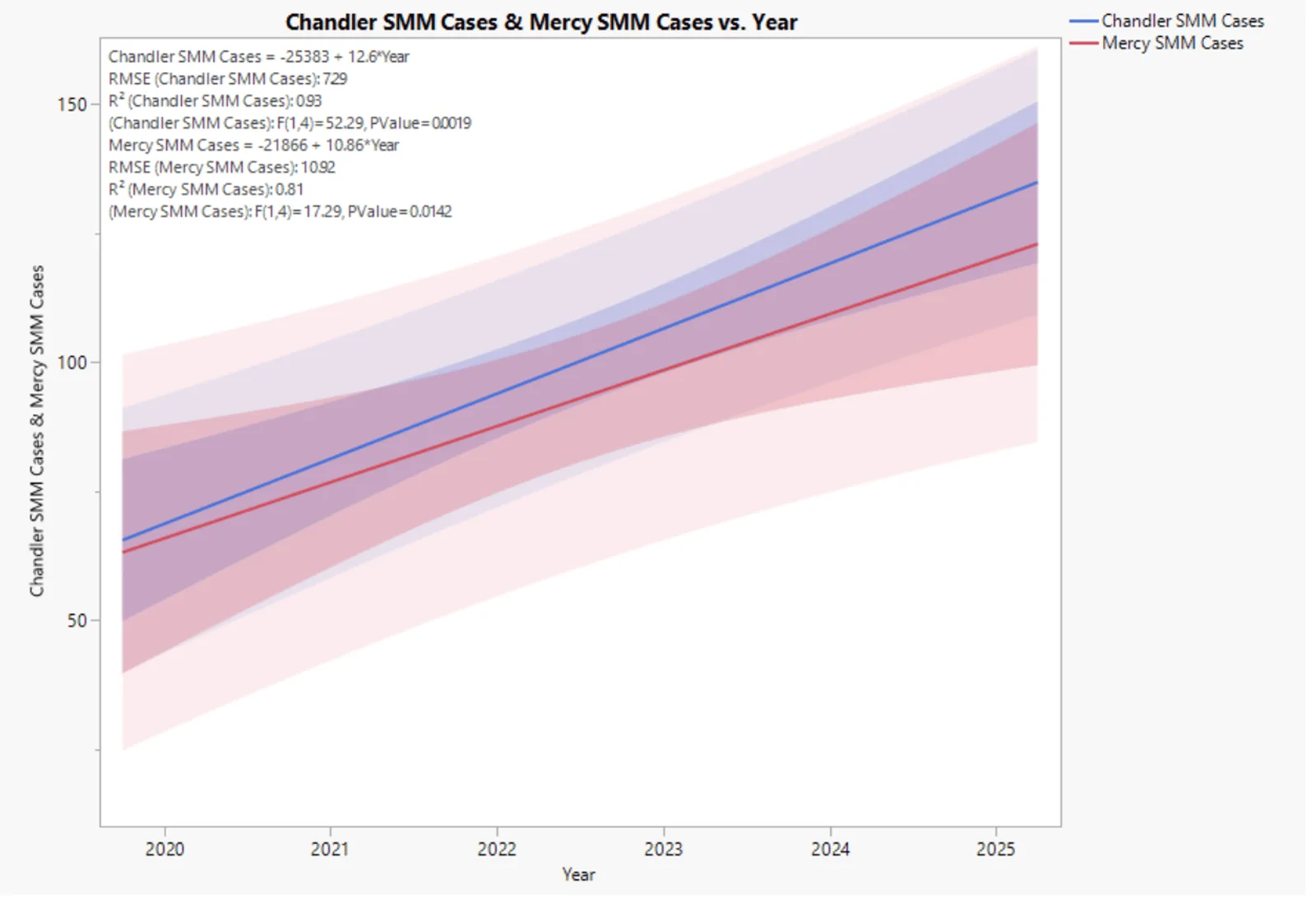
Linear regression of annual SMM case counts at CRMC and MGMC Linear regression demonstrated significant increasing trends in annual SMM case counts over time at both CRMC and MGMC. Annual SMM case counts increased by an estimated 12.6 cases per year at CRMC (R² = 0.93, p = 0.0019) and 10.9 cases per year at MGMC (R² = 0.81, p = 0.0142) SMM: severe maternal morbidity; CRMC: Chandler Regional Medical Center; MGMC: Mercy Gilbert Medical Center

SMM rates stratified by race/ethnicity

When stratified by race and ethnicity across both CRMC and MGMC, SMM rates increased across nearly all groups in 2025 compared with previous years (Figure 4). AI/AN non-Hispanic or Latino individuals had the highest SMM rate, which rose from 1.13% in 2020 to a peak of 6.17% in 2025 (15 SMM cases among 243 deliveries). Other racial and ethnic groups also saw their highest or near-highest SMM rates in 2025, including Asian non-Hispanic or Latino (4.61%), Black non-Hispanic or Latino (4.28%), Hispanic or Latino (3.65%), and White non-Hispanic or Latino individuals (3.11%).

**Figure 4:**
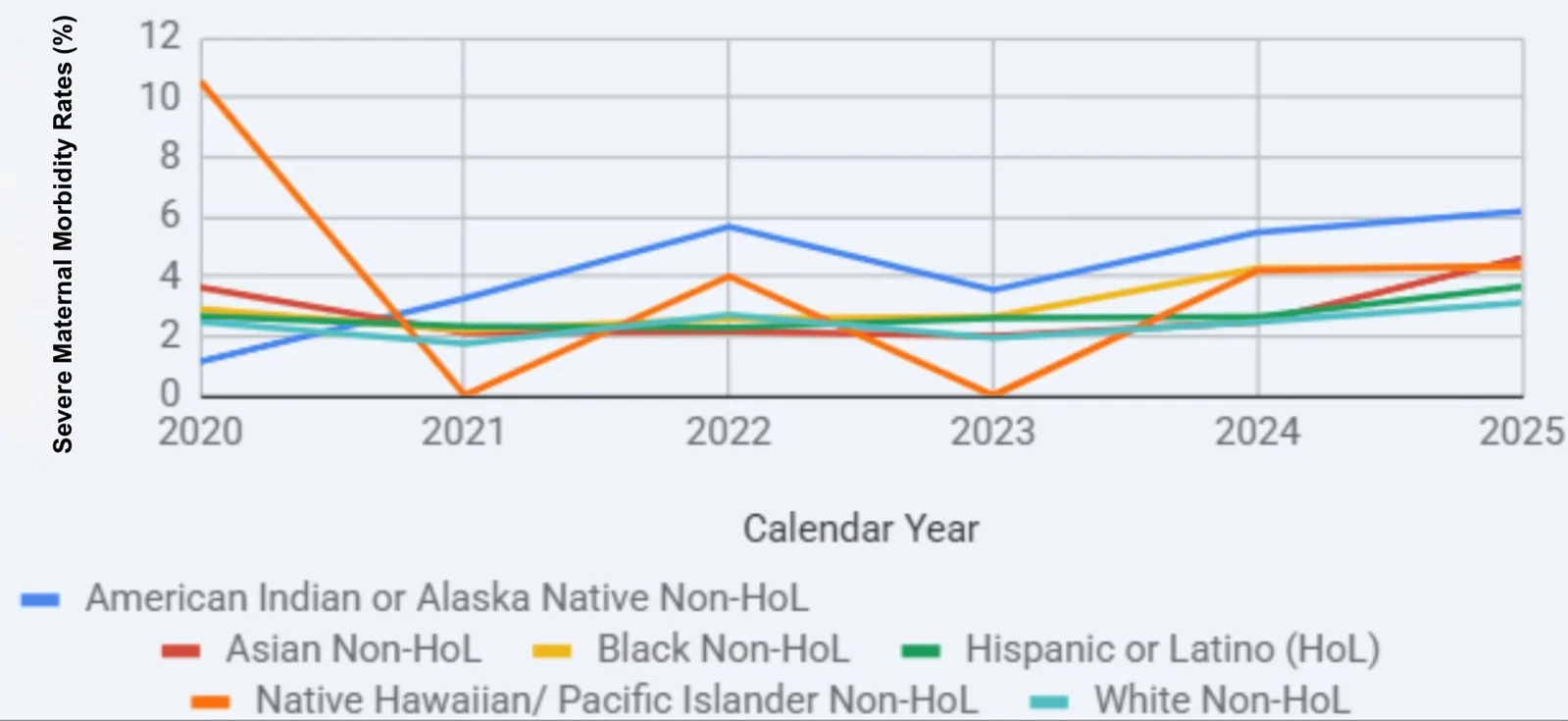
Trends in SMM rates by race and ethnicity, 2020-2025 Annual SMM rates across CRMC and MGMC were stratified by race and ethnicity to evaluate temporal trends from January 2020 through December 2025. SMM rates increased across nearly all racial and ethnic groups by 2025, with AI/AN non-Hispanic or Latino individuals demonstrating the greatest increase over the study period SMM: severe maternal morbidity; CRMC: Chandler Regional Medical Center; MGMC: Mercy Gilbert Medical Center; AI/AN: American Indian or Alaska Native

When data from CRMC and MGMC were combined across the 2020-2025 study period, cumulative SMM rates varied across racial and ethnic groups (Figure 5). AI/AN non-Hispanic or Latino individuals had the highest cumulative SMM rate at 4.12% (64/1,554 deliveries), followed by Native Hawaiian/Pacific Islander non-Hispanic or Latino individuals at 3.21% (6/187), Black non-Hispanic or Latino individuals at 3.17% (57/1,796), Asian non-Hispanic or Latino individuals at 2.81% (48/1,706), Hispanic or Latino individuals at 2.71% (159/5,871), and White non-Hispanic or Latino individuals at 2.38% (232/9,743). Compared with White non-Hispanic or Latino individuals, the cumulative SMM rate was 1.73 times higher among AI/AN individuals, 1.35 times higher among Native Hawaiian/Pacific Islander individuals, and 1.33 times higher among Black individuals.

**Figure 5:**
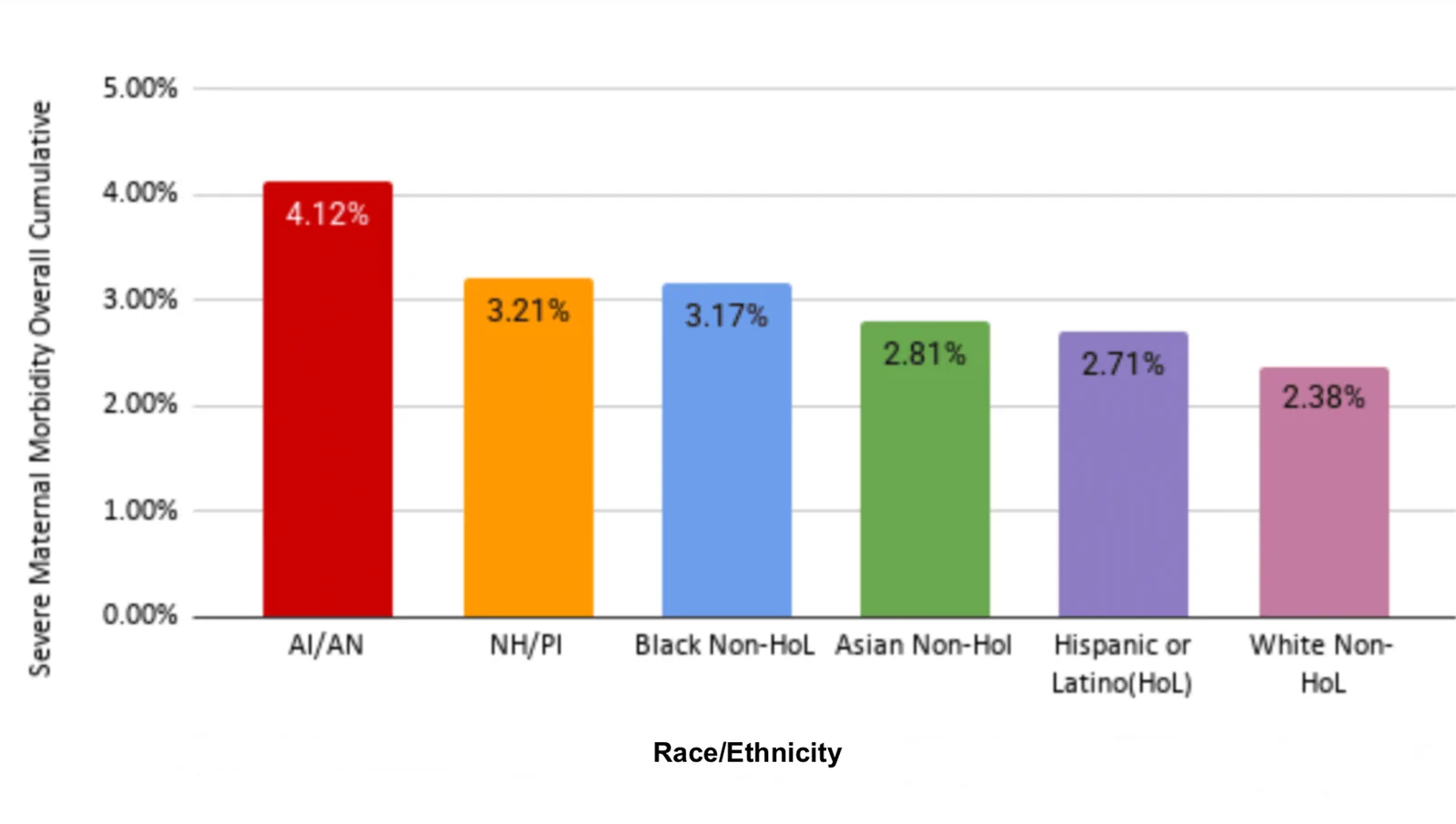
Cumulative SMM rates by race and ethnicity, 2020-2025 Cumulative SMM rates across CRMC and MGMC were calculated for each racial and ethnic group over the 2020-2025 study period. AI/AN non-HoL individuals had the highest cumulative SMM rate (4.12%), while White non-HoL individuals had the lowest (2.38%) SMM: severe maternal morbidity; CRMC: Chandler Regional Medical Center; MGMC: Mercy Gilbert Medical Center; AI/AN: American Indian or Alaska Native; NH/PL: Native Hawaiian or Other Pacific Islander; HoL: Hispanic or Latino

To further evaluate temporal trends among AI/AN individuals, linear regression was performed using annual SMM rates from 2020 to 2025 (Figure 6). SMM rates demonstrated a statistically significant increasing trend over the study period, with an estimated annual increase of 0.848 percentage points (p = 0.0415). The model explained approximately 68.7% of the variation in annual SMM rates (R² = 0.6866). Overall, the SMM rate increased from 1.13% in 2020 to 6.17% in 2025.

**Figure 6:**
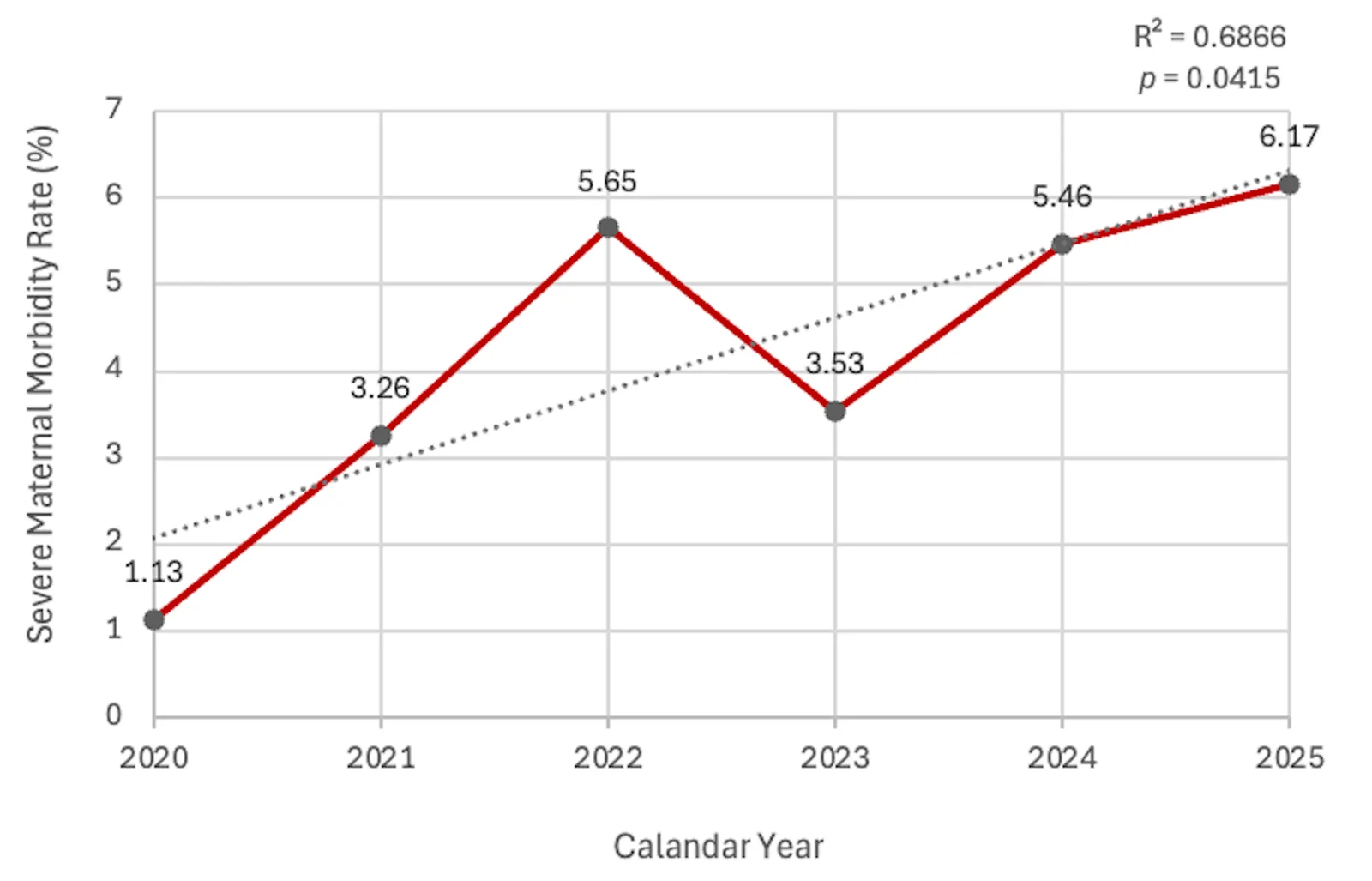
Linear regression of SMM rates among AI/AN individuals, 2020-2025 Annual SMM rates among AI/AN individuals across CRMC and MGMC are shown from 2020 through 2025. The dotted line represents the fitted linear regression, demonstrating a statistically significant increase of 0.848 percentage points per year (R² = 0.6866, p = 0.0415) SMM: severe maternal morbidity; AI/AN: American Indian or Alaska Native; CRMC: Chandler Regional Medical Center; MGMC: Mercy Gilbert Medical Center

## Discussion

This study shows a marked disparity in SMM among AI/AN birthing individuals receiving care in the East Valley of Arizona. Across both hospitals examined, AI/AN patients had the highest cumulative SMM rates compared with other racial and ethnic groups. Most notably, AI/AN patients showed a statistically significant increasing trend in SMM over the study period and were the only racial or ethnic group analyzed to show a significant increase over time. The increase from 1.13% in 2020 to 6.17% in 2025 represents a pronounced shift within a relatively short time frame and suggests that disparities in maternal outcomes within this population could be intensifying locally.

While previous research has documented racial and ethnic disparities in maternal outcomes across the United States, hospital-level data can provide additional insight into how these patterns play out within specific healthcare systems. National and statewide datasets often aggregate relatively small numbers of AI/AN patients, which can limit the ability to detect trends within particular communities. By examining outcomes within two hospitals serving the East Valley region, this study offers a more localized perspective on maternal morbidity affecting AI/AN patients receiving obstetric care in this area. Monitoring outcomes at this level may allow health systems to spot emerging disparities earlier and identify opportunities for targeted quality improvement, particularly when these hospitals serve as primary care sites for the population.

Clinical factors may contribute to the differences observed in this cohort. Pregnancy-related cardiovascular complications remain an important driver of severe maternal outcomes in the United States [6]. Conditions such as hypertensive disorders of pregnancy and cardiomyopathy account for a substantial proportion of severe maternal complications. AI/AN populations also experience higher rates of chronic health conditions, including hypertension, diabetes, obesity, and kidney disease, that are associated with increased risk of pregnancy complications [8,9]. These underlying health conditions may influence vulnerability to complications that meet criteria for SMM. While the present analysis focused primarily on trends over time, further examination of patient-level risk factors in the next phase of this study may help identify factors associated with the disparities observed here and inform future community-engaged research and interventions.

Differences in healthcare access and care delivery may also influence maternal outcomes. Previous work has highlighted barriers affecting many AI/AN communities, including geographic distance from obstetric services, limited availability of prenatal care providers, and fragmentation between tribal and non-tribal healthcare systems [11]. Such challenges may affect the timing of prenatal care initiation, continuity of care, and the management of high-risk conditions during pregnancy. In addition, culturally responsive care models and partnerships with tribal communities have been identified as important elements in improving maternal health outcomes among AI/AN populations [10]. Understanding how these structural and system-level factors interact with clinical risk factors will be important when interpreting disparities observed in hospital-based datasets.

The use of SPC methods is an additional strength of this study. SPC charts allowed for visualization of changes in monthly SMM case counts over time and helped identify patterns that might not be apparent from aggregate comparisons alone. Continuous monitoring of maternal outcomes using control charts and data-over-time approaches may support health systems in evaluating quality improvement initiatives and detecting shifts in maternal health indicators. The observed disparities are consistent with national and statewide data showing that AI/AN communities face disproportionate maternal health risks [1,4,5]. However, the statistically significant increasing trend in SMM observed among AI/AN individuals at the regional level deserves particular attention. These findings point to the need for further research on AI/AN maternal health disparities, particularly at the local level, to identify factors associated with these trends and inform future community-specific interventions.

Several limitations should be considered when interpreting these findings. First, the analysis was limited to two hospitals within a single healthcare system, which may limit generalizability to other regions or institutions. Second, the current analysis evaluated temporal trends using linear regression but did not adjust for potential confounding factors through multivariable analysis, such as maternal age, parity, socioeconomic conditions, insurance status, prenatal care, or preexisting medical conditions. The relatively small number of AI/AN deliveries in individual years may also contribute to variability in annual SMM rates, and confidence intervals were not calculated for race- and ethnicity-specific SMM estimates.

Additionally, changes in clinical documentation or coding practices over the study period could have influenced case identification. Because this study was primarily descriptive, the observed increase in SMM cannot determine whether the trend reflects changes in underlying morbidity or changes in patient volume, referral patterns, case mix, or detection over time. Finally, the use of electronic health record data raises the possibility of incomplete documentation or misclassification of race, ethnicity, or clinical diagnoses. Planned multivariable analyses and subgroup evaluations will provide additional insight into factors associated with severe maternal morbidity within this population.

Collaborations with local and regional AI/AN community partners are essential to mitigating existing barriers and ensuring culturally responsive care. Building on this necessity, the subsequent phase of this study will include subgroup analyses by age, access to prenatal care, socioeconomic factors, and comorbid conditions. These analyses are intended to identify factors associated with inequities in severe maternal morbidity among AI/AN individuals in the East Valley and to inform the development of targeted, community-specific interventions.

## Conclusions

This study presents a localized, hospital-based disparity analysis of SMM among AI/AN birthing individuals within the East Valley region of Arizona. The findings show that AI/AN patients at CRMC and MGMC had the highest cumulative SMM rates compared with other racial and ethnic groups analyzed. Most notably, AI/AN individuals were the only racial or ethnic group to show a statistically significant increase in SMM rates over the study period. These findings suggest that maternal health disparities within this population are not only persistent but could be worsening over time.

## References

[1] Chen Y, Shiels MS, Uribe-Leitz T, Molina RL, Lawrence WR, Freedman ND, Abnet CC (2025). Pregnancy-related deaths in the US, 2018-2022. JAMA Netw Open.

[2] MacDorman MF, Declercq E, Cabral H, Morton C (2016). Recent increases in the U.S. maternal mortality rate: disentangling trends from measurement issues. Obstet Gynecol.

[3] Fleszar LG, Bryant AS, Johnson CO (2023). Trends in state-level maternal mortality by racial and ethnic group in the United States. JAMA.

[4] Petersen EE, Davis NL, Goodman D (2019). Racial/ethnic disparities in pregnancy-related deaths - United States, 2007-2016. MMWR Morb Mortal Wkly Rep.

[5] (2026). Arizona Department of Health Services: Maternal mortalities and severe maternal morbidity in Arizona. https://www.azdhs.gov/documents/director/agency-reports/sb-1040-report-on-mmm-in-az.pdf.

[6] Collier AY, Molina RL (2019). Maternal mortality in the United States: updates on trends, causes, and solutions. Neoreviews.

[7] Heck JL, Jones EJ, Bohn D (2021). Maternal mortality among American Indian/Alaska Native women: a scoping review. J Womens Health (Larchmt).

[8] Celaya MF, Madhivanan P, McClelland J (2023). Individual and community-level risk factors for maternal morbidity and mortality among Native American women in the USA: protocol for systematic review. BMJ Open.

[9] Sharma G, Kelliher A, Deen J (2023). Status of maternal cardiovascular health in American Indian and Alaska Native individuals: a scientific statement from the American Heart Association. Circ Cardiovasc Qual Outcomes.

[10] Burns A, DeAtley T, Short SE (2023). The maternal health of American Indian and Alaska Native people: A scoping review. Soc Sci Med.

[11] Kozhimannil KB, Interrante JD, Henning-Smith C, Admon LK (2019). Rural-urban differences in severe maternal morbidity and mortality in the US, 2007-15. Health Aff (Millwood).

